# Investigating the relationship between body roundness index and low muscle mass based on a cross-sectional study: Focus on visceral adipose tissue

**DOI:** 10.1371/journal.pone.0326441

**Published:** 2025-08-19

**Authors:** Wei Huang, Huangyi Yin, Bijun Yang

**Affiliations:** 1 The First People’s Hospital of Chenzhou, Critical Care Medicine, Chenzhou, China; 2 The First Affiliated Hospital of Guangxi Medical University, Geriatric Endocrinology, Nanning, China; 3 The First Affiliated Hospital of Chongqing Medical University, Pulmonary and Critical Care Medicine, Chongqing, China; Dynamical Business & Science Society - DBSS International SAS, COLOMBIA

## Abstract

**Background:**

The relationship between body roundness index (BRI) and low muscle mass (LMM) remains unclear. This study investigated their association in American adults under 60 years.

**Methods:**

This secondary analysis utilized de-identified data from the National Health and Nutrition Examination Survey (NHANES, 2011–2018; n = 8,065 adults <60 years). Multivariable logistic regression evaluated associations between BRI and LMM, while multivariable linear regression assessed relationships between BRI and appendicular skeletal muscle mass (ASM)/BMI. Restricted cubic splines (RCS) tested nonlinearity, and receiver operating characteristic (ROC) curves compared BRI’s predictive performance against other body measurements indices. Finally, to assess the robustness of results, we conducted subgroup and sensivity analysis.

**Results:**

Each 1-unit BRI increase elevated LMM risk by 73% (OR=1.73, 95%CI = 1.61–1.86, *p* < 0.0001). Participants in the highest BRI quartile had 69-fold higher LMM odds versus the lowest quartile (OR=68.96, 95%CI = 33.62–141.47). RCS analysis revealed nonlinear positive BRI-LMM associations. Each10 units increase in BRI, ASM/ BMI decreased by 29% (β = −0.29,95% CI: −0.31, −0.28, *p* value < 0.0001). Participants in the highest BRI quartile had significantly lower ASM/ BMI levels, with corresponding β values of − 0.17. RCS analysis revealed nonlinear negative BRI- ASM/ BMI associations. When compared to other body measurements index, BRI shows good performance in identifying individuals at risk of LMM(AUC = 0.835).And sensitivity analyses confirmed robustness.

**Conclusion:**

Higher BRI may increase the risk of LMM in individuals under 60 years old among Americans, especially in men. BRI may serve as a supplementary indicator for identifying individuals at risk of LMM.

## Introduction

Sarcopenia is a disease marked by a reduction in the mass, strength, and overall function of skeletal muscle.[1,2]. With the increase of age, muscle fiber loss, neurodegeneration and protein dysfunction in skeletal muscle are aggravated, resulting in sarcopenia [3]. Based on a meta-analysis, sarcopenia influences more than 10% of global population over the age of 60 [4]. Sarcopenia can lead to a series of bad consequences, such as weakness, repeated falls, fractures, disabilities, poor quality of life, mortality and increased hospitalization rates [5–8], and in 2000 the direct medical cost of sarcopenia was as high as $ 18.5 billion in America [9].What is more, studies have shown that sarcopenia is related to many kinds diseases, including congestive heart failure, chronic obstructive pulmonary disease, nervous system diseases, diabetes mellitus, tumor-associated cachexia, inadequate nutrition and sarcopenic obesity [10–15]. Sarcopenia was once considered a geriatric disease, however, recent studies indicate that the loss of skeletal muscle related to sarcopenia typically begins at age 35 and progresses at a rate of approximately 1%−2% per year [16]. There existed 33% −66% of hygeian young female, in Japan, with presarcopenia [17,18]. Actively identifying the alterable risk factors of sarcopenia in young and middle-aged population, and finding effective screening tools and diagnostic methods are essential to reduce the risk of sarcopenia in the elderly in the future, slow disease progression, and decrease medical burden.

Globally, 39% of the adult population are overweight, 13% of which are classified as obese, and the number of obese people continues to rise [19,20]. The defining characteristic of sarcopenia is low muscle mass (LMM). And early-stage sarcopenia frequently manifests as LMM. Many researchers have also studied obesity and found an interaction between visceral adipose and LMM [21,22]. LMM decreases physical activity and energy consumption, and increases the risk of obesity [23]. In turn, visceral adipose triggers inflammation, which increases the risk of LMM [22]. The aggregation of visceral adipose in the body may refer to the pathophysiological and pathological process of sarcopenia or LMM [22]. However, traditional body measurements indicators, such as body mass index (BMI) and weight, may not be able to accurately determine the allocation of body fat, thus ignoring the adverse influence of visceral adipose tissue on the body. In order to overcome these limitations, researchers have exploited a new body measurement index, body roundness index (BRI), which takes into account waist circumference (WC) and height and can effectively identify visceral adipose volume [24].

However, whether BRI can effectively identify young and middle-aged population at high risk for LMM remains uncertain. After comprehensively considering the advantages of BRI and the important influence of visceral adipose tissue on LMM, we proposed a scientific hypothesis that the elevated BRI may be related to the increased risk of LMM. In order to fill the research gap in the relationship between BRI and LMM, this study analyzed data of participants from the National Health and Nutrition Survey (NHANES) to investigate the potential correlation between BRI and LMM, and the potential of BRI as a predictor of LMM, aiming to identify high-risk young and middle-aged population of sarcopenia early and provide theoretical support for the development of targeted prevention and treatment strategies.

## Methods

### Data source

The NHANES is a national survey of American residents sponsored by the National Center for Health Statistics (NCHS). In order to make the sample data representative of the health and nutrition data of the American population, a complex, multi-stage sampling method was used to collect data. The participants’ health and nutritional status were evaluated through interviews, physical examinations, laboratory tests and other items.

And all participants provided written informed consent when conducting a national survey in the United States. (https://www.cdc.gov/nchs/nhanes/index.htm). Since this study was a secondary analysis, ethical review and approval were exempted.

### Study participants

This study included 39,156 participants recruited by the NHANES between 2011 and 2018.

**Fig 1:**
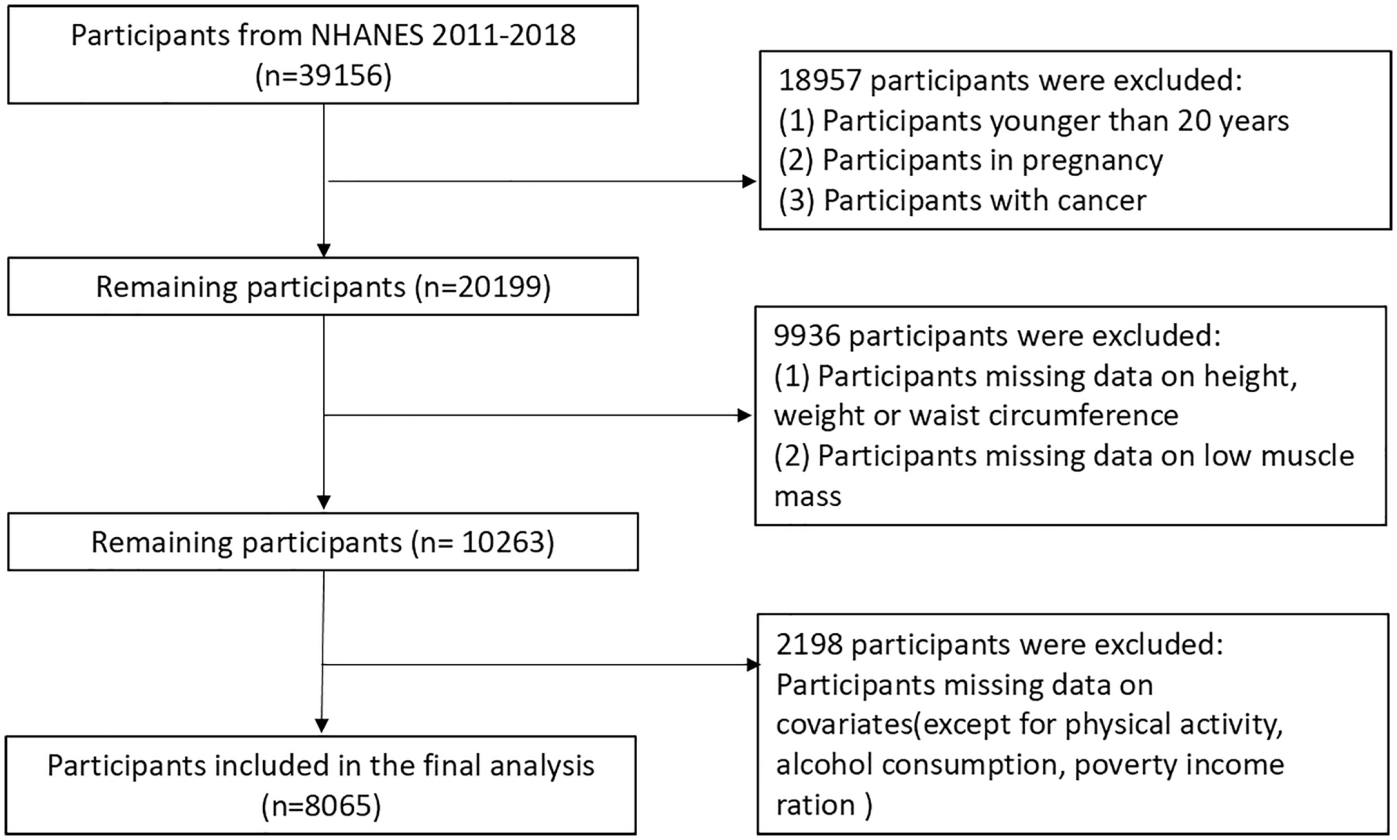
Flow diagram of participants selection.

As this is a secondary analysis, no direct recruitment or enrollment processes were conducted. We applied exclusion criteria to the existing dataset to enhance internal validity (Fig 1). It is noteworthy that dual-energy X-ray absorptiometry (DXA) examination of NHANES was only available for participants under the age of 60 and did not apply to pregnant women, individuals who were too heavy (over 136.4 kg), or too tall (over 192.5 cm). Considering that there were more missing values for some variables, such as physical activity (PA), alcohol consumption, poverty-to-income ratio (PIR), we classified the missing values of these variables into the “unknown” group.

### Evaluation of BRI and other obesity surrogate indices

Anthropometric measurements (such as weight, WC and height) were obtained in accordance with NHANES protocols, which are aligned with World Health Organization (WHO) standards for population health studies. We maintained original terminology for consistency with NHANES documentation and public health literature, rather than ISAK recommending the use of other terms (e.g., ‘body mass’ instead of ‘weight’).

BRI, first proposed by Thomas et al in 2013 [24], is a novel obesity indicator. BRI calculates body roundness based on the elliptical model of human body shape, and uses eccentricity to estimate the percentage of visceral fat to total fat. In addition to height, BRI also considers WC, so it can more fully reflect the distribution of visceral adipose tissue. In the Mobile Care Center (MEC), the participants’ height, WC and weight were tested by professional medical staff. Height was measured by standing height, usually using a fixed rangefinder or a wall-mounted rangefinder. To ensure measurement accuracy, participants must remain upright with their head, shoulders, and heels in contact with the vertical measuring plate. Weight was obtained using a calibrated digital floor scale. WC was typically assessed using a non-elastic tape, positioned midway between the iliac crest and the lower rib. The tape should be horizontal around the abdomen, and the measurement recorded at the end of exhalation. NHANES may employ multiple measurements and calculate the average to improve accuracy. This process required the participants to wear thin clothes and remove shoes and socks. The calculation process of body measurement indices, such as BMI, BRI and a body shape index (ABSI), involved in this study was as follows:


$$BMI=\frac{\;weight\;}{\;(height\;{)}^{2}}$$

$$BRI=364.2-365.5\times \sqrt{1-\frac{{\left(\frac{WC}{2\pi }\right)}^{2}}{(0.5\;height\;{)}^{2}}}$$

$$\;ABSI=\frac{WC}{(BMI{)}^{2/3}\times {\text{(}height\text{)}}^{1/2}}$$

### Diagnosis of low muscle mass

Although the gold standard for appendicular skeletal muscle mass (ASM) measurement is magnetic resonance imaging (MRI), appendage lean mass (ALM) by dual energy X-ray absorptiometry (DXA) is an affordable and practical alternative to ASM. McCarthy et al. developed an estimated SMM model with good performance through the ALM measured by DXA (ASM = 1.12 × ALM – 0.63) [25]. In this study, the diagnostic criteria for LMM were established according to the guidelines provided by the Foundation for the National Institutes of Health (FNIH) in 2014. Specifically, the sum of ASM was first assessed by DXA, and further divided by BMI. The final calculated ASM/BMI was used for the assessment of LMM. Participants were diagnosed as LMM if they fell below the ASM/BMI cut-off points of 0.512 and 0.789 for women and men, respectively [26].

### Covariates

In this study, we referred to previous studies and included the following potential covariates that may influence the relationship between BRI and LMM. Continuous variables included age, total energy intake, total protein intake, high-density lipoprotein cholesterol (HDL-C), total cholesterol (TC), creatinine, albumin, and uric acid. Total energy and protein intake was assessed based on the mean of the results of two dietary questionnaires, including the first on-site survey and the second telephone survey. Levels of HDL-C, TC, creatinine, albumin, and uric acid were further measured by taking blood samples from the participants at the MEC. Categorical variables were composed of race (non-hispanic whites, non-hispanic blacks, mexican americans, others), sex (male, female), smoking (previous smoking, current smoking, never smoking), PA (mild activity, high activity, unknown), education level (lower than high school, high school, higher than high school), drinking (never drinking, previous drinking, mild drinking, moderate drinking, heavy drinking, unknown), PIR (< 1.3,1.3–3.5, ≥ 3.5, unknown), marital status (married/living with partner, never married, widowed/ separated/ divorced), diabetes (yes, no), cardiovascular disease (CVD) (yes, no), hypertension (yes, no). Participants who indicated a personal history of diabetes and were taking hypoglycemic drugs were diagnosed with diabetes. The diagnosis of hypertension was determined through on-site detection of elevated blood pressure (a systolic reading exceeding 140 mmHg or a diastolic reading surpassing 90 mmHg), personal history of hypertension or taking antihypertensive medication to manage their blood pressure. Participants who self-reported a history of CVD were diagnosed with CVD.

### Statistical analysis

Analytical procedures adhered to STROSA reporting standards for secondary data [27].To increase the usability and representativeness of the study results, this study took into account the sample weights officially recommended by the NHANES in all the analytical procedures and followed the principle of minimum sample weights. When comparing the baseline characteristics of participants, T-test and Chi-square test were used for continuous variables and categorical variables, respectively, and results were shown as mean (standard deviation) and frequency (percentage). Post-hoc pairwise comparisons with Bonferroni adjustment were required to control the false positive rate for a P value of less than 0.05 for the chi-square test of multiple categorical variables. Adjusted significance thresholds were calculated as α = 0.05/number of comparisons. Before analysis, we assessed the collinearity of all covariates using the generalized variance-inflation factor (GVIF) in the “car” package, and covariates with GVIF^ (1/ (2*df)) <√10 were included in the final analysis. When evaluating the potential relationship between BRI and ASM/BMI and low muscle mass, we constructed models with three types of multivariate linear regression and logistic regression. Model 2 partially adjusted some covariates, such as sex, age, and race, while Model 1 did not adjust any covariates. Model 3 adjusted all potential covariates, specifically, including race, sex, age, smoking, PA, education level, drinking, PIR, marital status, diabetes, CVD, hypertension, total energy intake, total protein intake, HDL-C, TC, creatinine, albumin, uric acid. A post-hoc power analysis was conducted to evaluate the adequacy of our sample size using Power and Sample Size Calculation software (version 3.1.2). Using PS, a logistic regression model was specified with the following parameters: a two-sided alpha of 0.05 and 80% power threshold. Restricted cubic spline (RCS) analysis was utilized to estimate whether the correlation between BRI and LMM was nonlinear or linear. To evaluate the predictive performance of BRI for LMM risk identification, we generated receiver operating characteristic (ROC) curves and benchmarked its area under the curve (AUC) against other anthropometric indices. Participants were divided into different subgroups according to sex, age, race, CVD, diabetes, and hypertension. Subgroup analysis and interaction test were performed to investigate the robustness of these results. We conducted sensitivity analyses to assess the impact of missing data handling. Specifically, we re-analyzed the data by: 1) retaining the “unknown” group as originally specified, and 2) excluding participants with any missing values in PIR, PA, or alcohol consumption variables. P value less than 0.05 was deemed statistically significant and all analyses were carried out in R version 4.3.2.

## Results

### Baseline characteristics of participants

As illustrated in Table 1, after rigorous screening, a total of 8065 participants took part in our study,

**Table 1:** Weighted comparison of baseline characteristics.

Variables	Total (N = 8065)	Non-sarcopenia (N = 7389)	Sarcopenia (N = 676)	*P*-value
Age (years)	38.95(0.30)	38.65(0.31)	42.93(0.61)	< 0.0001
Sex (%)				0.300
Female	4115(49.47)	3776(49.68)	339(46.65)	
Male	3950(50.53)	3613(50.32)	337(53.35)	
Race (%)				< 0.0001*
Non-Hispanic Black	1716(11.29)	1671(11.88)	45(3.39)	
Non-Hispanic White	2831(59.41)	2660(60.64)	171(42.97)	
Mexican American	1201(11.54)	960(10.25)	241(28.72)	
Other	2317(17.76)	2098(17.22)	219(24.92)	
Marital status (%)				0.175
Never married	2130(25.98)	1994(26.30)	136(21.63)	
Separated	1075(12.40)	970(12.30)	105(13.84)	
Married	4860(61.62)	4425(61.40)	435(64.54)	
Education level (%)				< 0.0001*
Below high school	433(3.45)	327(2.86)	106(11.35)	
High school graduate	2678(30.77)	2402(30.00)	276(41.13)	
Above high school	4954(65.78)	4660(67.15)	294(47.52)	
PIR (%)				< 0.0001*
< 1.3	2348(22.38)	2101(21.70)	247(31.60)	
1.3-3.5	2713(32.27)	2485(32.06)	228(35.13)	
≥ 3.5	2396(39.17)	2258(40.25)	138(24.72)	
Unknown	596(6.17)	535(6.00)	61(8.55)	
PA (%)				< 0.0001*
Low	1002(12.03)	918(11.96)	84(13.00)	
High	5539(72.01)	5166(73.07)	373(57.81)	
Unknown	1512(15.96)	1295(14.98)	217(29.19)	
Smoking (%)				0.570
Never	4996(60.47)	4562(60.31)	434(62.63)	
Former	1331(18.85)	1210(18.82)	121(19.25)	
Now	1726(20.68)	1607(20.87)	119(18.13)	
Drinking (%)				< 0.0001*
Never	998(9.72)	856(9.06)	142(18.50)	
Former	687(8.18)	606(7.87)	81(12.34)	
Mild	2548(32.27)	2377(32.99)	171(22.50)	
Moderate	1424(19.13)	1346(19.69)	78(11.58)	
Heavy	1869(25.22)	1722(24.97)	147(28.57)	
Unknown	527(5.48)	472(5.41)	55(6.50)	
Protein intake (g/day)	85.20(0.60)	85.68(0.61)	78.69(1.55)	< 0.0001
Energy intake (Kcal/day)	2162.77(15.01)	2174.60(15.29)	2004.32(41.73)	< 0.001
Diabetes (%)				< 0.0001
No	7446(94.18)	6884(94.95)	562(83.87)	
Yes	619(5.82)	505(5.05)	114(16.13)	
Hypertension (%)				< 0.0001
No	5831(74.47)	5427(75.85)	404(56.09)	
Yes	2234(25.53)	1962(24.15)	272(43.91)	
CVD (%)				< 0.001
No	7795(97.08)	7166(97.42)	629(92.48)	
Yes	270(2.92)	223(2.58)	47(7.52)	
TC (mmol/l)	4.96(0.02)	4.96(0.02)	5.06(0.05)	0.055
HDL-C (mmol/l)	1.36(0.01)	1.37(0.01)	1.24(0.02)	< 0.0001
Uric acid (umol/l)	317.39(1.57)	316.40(1.65)	330.66(4.64)	0.005
Creatinine (umol/l)	75.93(0.43)	76.44(0.43)	68.99(0.96)	< 0.0001
Albumin (g/l)	43.39(0.09)	43.50(0.09)	41.90(0.20)	< 0.0001
Weight (kg)	81.98(0.36)	81.33(0.35)	90.64(1.32)	< 0.0001
WC (cm)	97.23(0.34)	96.22(0.31)	110.80(0.96)	< 0.0001
BMI (kg/m^2^)	28.66(0.14)	28.16(0.12)	35.29(0.40)	< 0.0001
ABSI	0.08(0.00)	0.08(0.00)	0.08(0.00)	< 0.0001
BRI	5.08(0.05)	4.87(0.04)	7.91(0.13)	< 0.0001

* Significant after Bonferroni correction.

Categorical variables were assessed with the chi-square test, whereas continuous variables were compared using the t-test. And results were shown as mean frequency (percentage) and (standard deviation).

Categorical variables: sex, race, marital status, education level, PIR, PA, smoking, drinking, diabetes, hypertension, CVD.

Continuous variables: age, protein intake, energy intake, TC, HDL-C, uric acid, creatinine, albumin, weight, WC, BMI, ABSI, BRI.

PIR, poverty income ratio; PA, physical activity; CVD, cardiovascular disease; TC, total cholesterol; HDL-C, high-density lipoprotein cholesterol; WC, waist circumference; BMI, body mass index; ABSI, a body shape index; BRI, body roundness index.

with an average age of 38.95 (0.30) and a male proportion of 50.53%. The mean BRI of all participants was 5.08(0.05), and the number of participants with LMM were 676. In comparison with the non-sarcopenic participants, LMM participants were older, more Mexican American, and heavier drinkers, had lower levels of education and economic income, were less physically active, and tended to have diabetes, hypertension, and CVD. In terms of diet, LMM participants preferred to consume more energy and less protein. In terms of laboratory tests, the comparison of HDL-C, uric acid, creatinine and albumin between the two groups was also statistically significant. Importantly, obesity-related parameters, including weight, WC, BMI, ABSI, and BRI, were also demonstrably greater in LMM participants than in non-LMM participants.

### The relationship between BRI and low muscle mass and ASM/ BMI

After fully considering potential confounding factors, three multivariate logistic regression models were utilized to explore the relationship between BRI and low muscle mass. We tested for collinearity of all covariates. Covariates with GVIF^(1/(2*Df))<√10 were included in the final study. When BRI was assessed as a continuous variable, BRI was noticeable positively correlated with the incidence of LMM in all three models. In Model 3, for each 1-unit rise in the BRI, the likelihood of developing LMM escalated by 73%. (OR,1.73;95% CI,1.61–1.86; **p* *< 0.0001).

**Fig 2:**
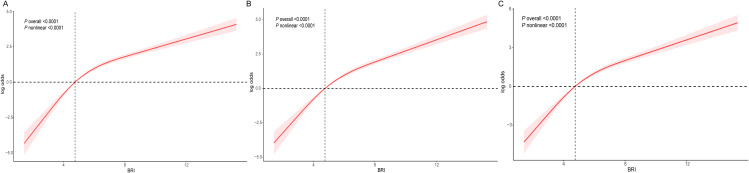
Weighted RCS curves for association between BRI and the prevalence of low muscle mass. Adjusted for gender, age, race, marital status, PIR, smoking status, alcohol consumption, education level, PA, hypertension, CVD, diabetes, TC, HDL-C, creatinine, uric acid, albumin, energy intake, protein intake. The red shaded areas represent the 95% CI.

In addition, when BRI was divided into quartiles (Q1, Q2, Q3, Q4), participants in the fourth quartile demonstrated a substantially elevated risk of LMM than those in the first quartile, with an OR value of 68.96 (OR,68.96;95% CI,33.62–141.47; *p* < 0.0001). As illustrated in Fig 2 A−C, the RCS analysis manifested nonlinear positive correlation between the BRI and LMM. The comprehensive results of the logistic regression analysis were thoroughly shown in Table 2.

**Table 2:** Weighted logistic regression for association between BRI and low muscle mass.

Exposures	Model 1 [OR (95% CI) *P*-value]	Model 2 [OR (95% CI) *P*-value]	Model 3 [OR (95% CI) *P*-value]
BRI (Continuous)	1.62(1.55,1.69) <0.0001	1.70(1.61,1.80) <0.0001	1.73(1.61, 1.86) <0.0001
BRI (Quartiles)			
Q1 (≤3.54)	ref	ref	ref
Q2 (3.54–4.76)	6.58(3.09, 14.03) <0.0001	5.29(2.46, 11.41) <0.0001	5.73(2.64, 12.44) <0.0001
Q3 (4.76–6.34)	21.88(10.19, 47.00) <0.0001	16.34(7.42, 35.97) <0.0001	18.56(8.59, 40.14) <0.0001
Q4 (>6.34)	78.52(38.12,161.72) <0.0001	68.91(32.55,145.87) <0.0001	68.96(33.62,141.47) <0.0001
*P* for trend	<0.0001	<0.0001	<0.0001

Model 1: Adjusted for no variables.

Model 2: Adjusted for race, sex, and age.

Model 3: Adjusted for sex, age, race, marital status, PIR, smoking status, alcohol consumption, education level, PA, hypertension, CVD, diabetes, TC, HDL-C, creatinine, uric acid, albumin, energy intake, protein intake.

BRI, body roundness index; OR, odds ratio.

**Fig 3:**
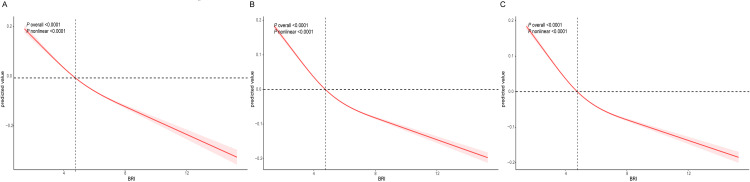
Weighted RCS curves for association between BRI and the prevalence of ASM/ BMI. Adjusted for gender, age, race, marital status, PIR, smoking status, alcohol consumption, education level, PA, hypertension, CVD, diabetes, TC, HDL-C, creatinine, uric acid, albumin, energy intake, protein intake. The red shaded areas represent the 95% CI.

In addition, we conducted a multivariate linear regression analysis to explore the correlation between BRI and ASM/BMI. As shown in Table 3, BRI and ASM/ BMI maintained a negative dose-response relationship in all three models. After considering all confounding factors, for every 10 units increase in BRI, ASM/ BMI decreased by 29% (β = −0.29,95% CI: −0.31, −0.28, *p* value < 0.0001). Compared with individuals with the lowest quartile of BRI, participants with the second, third, and fourth quartiles had significantly lower ASM/ BMI levels, with corresponding β values of − 0.07, − 0.12, and − 0.17, respectively (Q2: β = − 0.07,95% CI: − 0.08, − 0.06, *p* value < 0.0001; Q3: β = −0.12,95% CI: −0.13, −0.11, *p* value < 0.0001; Q4: β = −0.17,95% CI: −0.18, −0.16, *p* value < 0.0001). RCS analysis showed that the negative dose-response relationship between BRI and ASM/ BMI was nonlinear (Fig 3A−C).

**Table 3:** Weighted Linear Regression Analysis of BRI and ASM/BMI.

Exposures	Model 1 [β (95% CI) *P*-value]	Model 2 [β (95% CI) *P*-value]	Model 3 [β (95% CI) *P*-value]
BRI (Per 10 units increase)	−0.40(−0.43,-0.38) <0.0001	−0.31(−0.33,-0.30) <0.0001	−0.29(−0.31,-0.28) <0.0001
BRI (Quartiles)			
Q1 (≤3.54)	ref	ref	ref
Q2 (3.54–4.76)	−0.07(−0.09,-0.05) <0.0001	−0.07(−0.08,-0.06) <0.0001	−0.07(−0.08,-0.06) <0.0001
Q3 (4.76–6.34)	−0.13(−0.15,-0.11) <0.0001	−0.12(−0.13,-0.11) <0.0001	−0.12(−0.13,-0.11) <0.0001
Q4 (>6.34)	−0.24(−0.25,-0.22) <0.0001	−0.19(−0.20,-0.18) <0.0001	−0.17(−0.18,-0.16) <0.0001
*P* for trend	<0.0001	<0.0001	<0.0001

Model 1: Adjusted for no variables.

Model 2: Adjusted for race, sex, and age.

Model 3: Adjusted for sex, age, race, marital status, PIR, smoking status, alcohol consumption, education level, PA, hypertension, CVD, diabetes, TC, HDL-C, creatinine, uric acid, albumin, energy intake, protein intake.

BRI, body roundness index; ASM/BMI, appendicular skeletal muscle mass adjusted by body mass index.

The post-hoc power analysis demonstrated that with 8,065 participants (including 676 cases), the study achieved 99.3% statistical power (95% CI: 98.7–99.8%) to detect an odds ratio of 1.73 per BRI unit increase, using F-adjusted likelihood ratio test for complex samples. This exceeds the conventional 80% power threshold, confirming the sample size was sufficient to identify clinically meaningful associations.

### Comparing the predictive performance of different surrogate indices for low muscle mass

**Fig 4:**
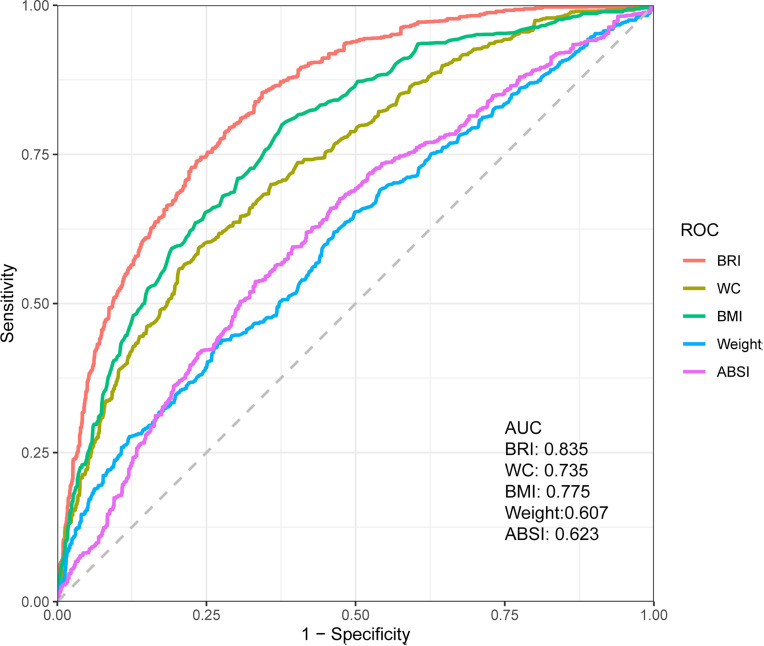
Weighted ROC curves for BRI, WC, BMI, Weight and ABSI.

In order to clarify the advantage of BRI in identifying LMM individuals, this study further plotted the ROC curve to compare the predictive performance of BRI with other anthropometric indices for LMM. As shown in Fig 4, BRI showed good performance in identifying individuals at risk of LMM, with an obtaining AUC value of 0.835. In addition, the AUC values of other anthropometric indices were 0.775, 0.735, 0.623 and 0.607 from high to low, corresponding to BMI, WC, ABSI and weight respectively.

### Subgroup and sensitivity analyses

**Fig 5:**
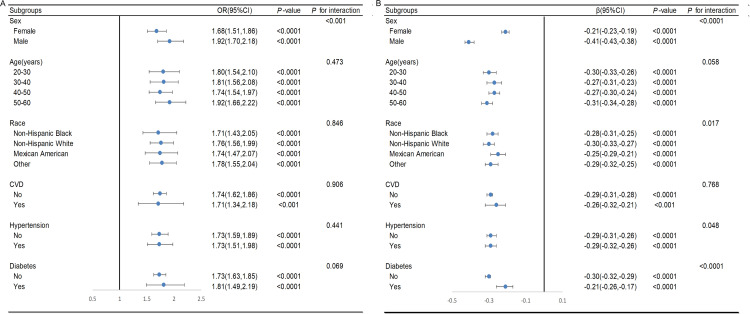
Subgroup analysis and interaction test of the association between BRI and the prevalence of low muscle mass and ASM/ BMI. Adjusted for gender, age, race, marital status, PIR, smoking status, alcohol consumption, education level, PA, hypertension, CVD, diabetes, TC, HDL-C, creatinine, uric acid, albumin, energy intake, protein intake, except the subgroup factors themselves.

As shown in Fig 5, we divide the participants into different subgroups to further test the robustness of these results. Participants were divided into different subgroups according to sex, age, race, CVD, diabetes, and hypertension. subgroup analysis and interaction tests were performed to investigate the robustness of these results. The correlation between BRI and LMM was positive in different subgroups and BRI had no marked interaction with age, race, CVD, hypertension and diabetes (*p* for interaction>0.05), which manifested the relationship between BRI and LMM remaining stable at these subgroups. However, there existed a notable interaction between BRI and sex (*p* for interaction <0.05), and the positive relationship between BRI and LMM was more obvious in men (OR,1.92;95% CI,1.70–2.18; *p* < 0.0001). In addition, BRI was negatively correlated with ASM/ BMI in different subgroups. There was a significant interaction between BRI and gender, race, diabetes and hypertension (*p* for interaction < 0.05).

Finally, we performed a sensitivity analysis to exclude all participants with missing PA, PIR, and alcohol intake. The results were also consistent with the main findings (Supplementary Tables 1 and 2).

## Discussion

In our study, a large high-quality sample of the NHANES database was applied to assess the relationship between BRI and LMM for the first time, and the results are worthy of attention.

We found a significant positive correlation between BRI and the prevalence of LMM, and a negative dose-response relationship with ASM/BMI levels, especially in men. We found that BRI demonstrated good performance in identifying individuals at risk of LMM, outperforming other anthropometric indices such as WC, BMI, and ABSI.

As the global population ages, it is anticipated that the prevalence of sarcopenia patients will rise from 50 million to over 200 million within the next four decades, which will greatly increase the medical burden [28]. Studies indicate that if the prevalence of sarcopenia were reduced by 10%, health care costs in the United States could be reduced by $1.1 billion [9]. Sarcopenia was considered an age-related disease that usually only gets attention in the older population [29]. However, there existed 33% −66% of hygeian young female, in Japan, with presarcopenia [17,18]. And sarcopenia affected 5–10% of young and middle-aged population in America [30]. Life-cycle model shows that muscle mass and strength peaks early in life and then gradually declines in middle-to-late life [29,31]. The muscle loss in early period can lead to a considerable decrease in muscle mass and strength in later period [32]. If LMM occurs in young and middle-aged population, because of its longer duration, it may lead to more serious adverse outcomes, such as severe clinical manifestations, poor prognosis, and higher hospitalization costs [9,29,33]. Therefore, LMM should be more emphasized from a young age to give due attention, appropriate prevention and timely treatment. Unfortunately, now most preventive epidemiological studies of LMM focus on slowing the decline in muscle mass and strength in old age [29]. Too much attention is paid to elderly population, while the impact of LMM on young and middle-aged population is ignored. The effect of obesity on LMM has attracted increasing scholarly attention. Studies have found that LMM is closely related to obesity. With the increase of age, the loss of skeletal muscle mass, the increase of fat mass, and the redistribution of fat also occur, which is manifested by fat transfer from the subcutaneous area to the abdominal cavity (visceral adipose) [34–36]. Elevated visceral adipose increases the risk of LMM [37]. It is impossible to differentiate between body fat mass and body fat-free mass via BMI [38]. Because BMI does not consider the decrease of muscle mass with age, it is inaccurate to utilize BMI as an indicator to verify obesity-related complications [39]. WC is a simple indicator to evaluate visceral fat, which can make up for the lack of BMI to reflect body fat distribution, but it cannot avoid the defect of tall people having larger WC [30,40]. BRI, a new obesity index first proposed by Thomas et al. in 2013, comprehensively reflects body roundness and visceral adipose distribution based on height and WC calculation [24], which is more advantageous than single body measurement indicators, such as WC, BMI and weight. Several studies have demonstrated that BRI has certain advantages in terms of accuracy in the diagnosis of diseases, such as diabetes and prediabetes, metabolically obese normal weight (MONW), gallstones [41–43]. Therefore, this study focused on the young and middle-aged population to explore the correlation between BRI and LMM. We found that BRI was associated with an increased risk of LMM and showed good performance in identifying at-risk individuals compared to traditional indices like WC, BMI, and ABSI.

BRI, as a surrogate measure of visceral adipose load, integrates WC and height to quantify visceral adipose distribution. Elevated BRI values reflect higher visceral adiposity, which is associated with LMM risk through multiple mechanistic pathways. Adipose tissue is transferred from subcutaneous to visceral sites, resulting in a disparity between pro-inflammatory adipokines and anti-inflammatory myokines [44]. The aggregation of adipose tissue and activation of macrophages causes raised secretion of pro-inflammatory cytokines, e.g., interleukin (IL)-1, IL-6 and tumor necrosis factor-α (TNF-α) [45,46].What is more, adipose tissue secretes pro-inflammatory adipokines that contribute to lipotoxicity in skeletal muscle, which in turn promotes the development of LMM [47].Increased leptin, an adipocyte hormone, may lead to leptin resistance and a decrease in fatty acid oxidation in the muscle, causing ectopic fat deposition in skeletal muscle and other tissues, resulting in muscle atrophy [48]. Studies also have found that increased visceral adipose is strongly associated with IR [49]. IR is considered a crucial factor in the progression of LMM.IR can promote the development of LMM by increasing muscle protein degradation [50], decreasing protein synthesis, up-regulating FoxO family expression [51,52], and activating autophagy in skeletal muscle cells [53].

Interestingly, we also found that BRI was notably more related to LMM in men than in women. Our study is consistent with Kristina et al. ‘s study, which suggested that men may experience more severe skeletal muscle mass loss and dysfunction than women [54]. In addition, under the same degree of muscle loss, men may face a higher risk of disease or death than women [55,56]. These sex differences may be relevant to hormonal status (testosterone, luteinizing hormone) in men [57,58]. From the age of 30 in men, the level of this hormone drops by 1% annually, and bioavailable testosterone drops by 2% annually [59]. The aging-related decrease in testosterone is paralleled by a reduction in body fat-free mass and a rise in body fat mass, resulting in sarcopenic obesity, which explains the results of this study [60].

### Limitations and strengths

This study assesses the potential relationship between BRI and LMM, partially explains the important role of visceral adipose tissue in the pathologic and physiopathologic process of LMM, and helps to develop individualized diagnosis and treatment of LMM. In our study, participants were recruited from the NHANES, a high-quality public database, ensuring sufficient sample size and data authenticity. In addition, we considered the sample weight of NHANES to ensure that the results were applicable to Americans under the age of 60. However, there are still several limitations worth noting. First, although we have considered many covariates, there are still other potential confounding factors that cannot be avoided to affect the relationship between BRI and LMM. Secondly, the population of this study is limited to American residents, and whether the conclusion can be extended to other populations still needs further verification. What is more, we explored the association between BRI and LMM based on the cross-sectional study, and in order to identify the causality between BRI and LMM, it is necessary to perform cohort studies. Finally, at present, the diagnostic criteria for LMM are still not uniform, and different diagnostic criteria may also lead to inconsistent results.

## Conclusion

In short, our study found that higher BRI may increase the risk of LMM in individuals under 60 years old among Americans, especially in men. BRI may serve as a supplementary tool to existing diagnostic modalities. We advocate actively controlling visceral adipose and focusing on BRI index, especially in the male population, to decrease the risk of LMM. Future studies could harness machine learning algorithms to integrate multi-dimensional biomarkers (e.g., BRI, metabolic profiles) for enhancing LMM risk prediction and identifying novel mechanistic pathways across diverse populations.

## Supporting information

S1 Table Weighted logistic regression for association between BRI and low muscle mass (excluding participants with missing data on PA, PIR, and alcohol intake). Model 1: Adjusted for no variables. Model 2: Adjusted for race, gender, and age. Model 3: Adjusted for gender, age, race, marital status, PIR, smoking status, alcohol consumption, education level, PA, hypertension, CVD, diabetes, TC, HDL-C, creatinine, uric acid, albumin, energy intake, protein intake. BRI: body roundness index; OR: odds ratio.
(DOCX)

S2 Table Weighted linear regression analysis of BRI and ASM/BMI (excluding participants with missing data on PA, PIR, and alcohol intake). Model 1: Adjusted for no variables. Model 2: Adjusted for race, gender, and age. Model 3: Adjusted for gender, age, race, marital status, PIR, smoking status, alcohol consumption, education level, PA, hypertension, CVD, diabetes, TC, HDL-C, creatinine, uric acid, albumin, energy intake, protein intake. BRI: body roundness index; ASM/BMI: appendicular skeletal muscle mass adjusted by body mass index.
(DOCX)

S1 Data Minimal data set. (XLSX)

## Abbreviations

BRI: body roundness index
NHANES: National Health and Nutrition Survey
RCS: restricted cubic spline
ROC: receiver operating characteristic
LMM: low muscle mass
BMI: body mass index
WC: waist circumference
NCHS: National Center for Health Statistics
DXA: dual-energy X-ray absorptiometry
PA: physical activity
PIR: poverty-to-income ratio
MEC: Mobile Care Center
ABSI: a body shape index
FNIH: National Institutes of Health Foundation
ASM: appendicular skeletal muscle mass
HDL-C: high-density lipoprotein cholesterol
TC: total cholesterol, CVD, cardiovascular disease
AUC: area under the curve
IR: insulin resistance
MONW: metabolically obese normal weight
IL: interleukin
TNF-α: tumor necrosis factor-α
BIA: bioelectrical impedance analysis
CT: computed tomography
MRI: magnetic resonance imaging.
