# Supplementary material for: Investigating the relationship between body roundness index and low muscle mass based on a cross-sectional study: Focus on visceral adipose tissue
Source: PLoS One. 2025 Aug 19;20(8):e0326441. doi: 10.1371/journal.pone.0326441 (PMC12364339; doi:10.1371/journal.pone.0326441)
Supplement: S1 Table — Model 1: Adjusted for no variables. Model 2: Adjusted for race, gender, and age. Model 3: Adjusted for gender, age, race, marital status, PIR, smoking status, alcohol consumption, education level, PA, hypertension, CVD, diabetes, TC, HDL-C, creatinine, uric acid, albumin, energy intake, protein intake. BRI: body roundness index; OR: odds ratio. (DOCX) [file pone.0326441.s001.docx]

**S1 Table. Weighted logistic regression for association between BRI and low muscle mass (excluding participants with missing data on PA, PIR, and alcohol intake).**

| Exposures | Model1  [OR (95% CI) *P*-value] | Model2  [OR (95% CI) *P*-value] | Model3  [OR (95% CI) *P*-value] |
| --- | --- | --- | --- |
| BRI (Continuous) | 1.65(1.57,1.74) <0.0001 | 1.73(1.64,1.83)  <0.0001 | 1.73(1.58,1.90)  <0.0001 |
| BRI (Quartiles) |  |  |  |
| Q1 (≤3.42) | ref | ref | ref |
| Q2 (3.42-4.63) | 9.02( 2.87, 28.33) <0.001 | 7.33( 2.31, 23.25) 0.001 | 7.59( 2.22, 26.00) 0.002 |
| Q3 (4.63-6.14) | 33.29(11.72, 94.54) <0.0001 | 24.58( 8.44, 71.61) <0.0001 | 24.66( 8.23, 73.92) <0.0001 |
| Q4 (>6.14) | 125.23(46.37,338.22) <0.0001 | 103.91(38.20,282.61) <0.0001 | 98.95(34.17,286.52) <0.0001 |
| *P* for trend | <0.0001 | <0.0001 | <0.0001 |

Model 1: Adjusted for no variables.

Model 2: Adjusted for race, gender, and age.

Model 3: Adjusted for gender, age, race, marital status, PIR, smoking status, alcohol consumption, education level, PA, hypertension, CVD, diabetes, TC, HDL-C, creatinine, uric acid, albumin, energy intake, protein intake.

BRI: body roundness index; OR: odds ratio.
