# Supplementary material for: Investigating the relationship between body roundness index and low muscle mass based on a cross-sectional study: Focus on visceral adipose tissue
Source: PLoS One. 2025 Aug 19;20(8):e0326441. doi: 10.1371/journal.pone.0326441 (PMC12364339; doi:10.1371/journal.pone.0326441)
Supplement: S2 Table — Model 1: Adjusted for no variables. Model 2: Adjusted for race, gender, and age. Model 3: Adjusted for gender, age, race, marital status, PIR, smoking status, alcohol consumption, education level, PA, hypertension, CVD, diabetes, TC, HDL-C, creatinine, uric acid, albumin, energy intake, protein intake. BRI: body roundness index; ASM/BMI: appendicular skeletal muscle mass adjusted by body mass index. (DOCX) [file pone.0326441.s002.docx]

**S2 Table. Weighted linear regression analysis of BRI and ASM/BMI (excluding participants with missing data on PA, PIR, and alcohol intake).**

| Exposures | Model1  [β (95% CI) *P*-value] | Model2  [β (95% CI) *P*-value] | Model3  [β (95% CI) *P*-value] |
| --- | --- | --- | --- |
| BRI (Per 10 units increase) | -0.41(-0.44,-0.38) <0.0001 | -0.33(-0.35,-0.31) <0.0001 | -0.30(-0.33,-0.28) <0.0001 |
| BRI (Quartiles) |  |  |  |
| Q1 (≤3.42) | ref | ref | ref |
| Q2 (3.42-4.63) | -0.06(-0.08,-0.04) <0.0001 | -0.07(-0.08,-0.06) <0.0001 | -0.07(-0.08,-0.06) <0.0001 |
| Q3 (4.63-6.14) | -0.12(-0.14,-0.10) <0.0001 | -0.13(-0.14,-0.11) <0.0001 | -0.12(-0.13,-0.10) <0.0001 |
| Q4 (>6.14) | -0.23(-0.25,-0.21) <0.0001 | -0.20(-0.21,-0.18) <0.0001 | -0.18(-0.19,-0.16) <0.0001 |
| *P* for trend | <0.0001 | <0.0001 | <0.0001 |

Model 1: Adjusted for no variables.

Model 2: Adjusted for race, gender, and age.

Model 3: Adjusted for gender, age, race, marital status, PIR, smoking status, alcohol consumption, education level, PA, hypertension, CVD, diabetes, TC, HDL-C, creatinine, uric acid, albumin, energy intake, protein intake.

BRI: body roundness index; ASM/BMI: appendicular skeletal muscle mass adjusted by body mass index.
